# Sexual Health of Polish Athletes with Disabilities

**DOI:** 10.3390/ijerph120707417

**Published:** 2015-06-30

**Authors:** Ryszard Plinta, Joanna Sobiecka, Agnieszka Drosdzol-Cop, Agnieszka Nowak-Brzezińska, Agnieszka Kobiołka, Violetta Skrzypulec-Plinta

**Affiliations:** 1 School of Health Sciences in Katowice, Medical University of Silesia, Katowice, Department of Adapted Physical Activity and Sport, Chair of Physiotherapy, Medyków 12, 40-752 Katowice, Poland; E-Mail: ryszardplinta@wp.pl; 2 Faculty of Motor Rehabilitation, University School of Physical Education, Al. Jana Pawła II 78, 31-571 Krakow, Poland; E-Mail: J.W.Sobiecka@interia.pl; 3 School of Health Sciences in Katowice, Medical University of Silesia, Katowice, Chair of Woman’s Health, Medyków 12, 40-752 Katowice, Poland; E-Mails: agakobiolka@o2.pl (A.K.) skrzypulec-plinta@o2.pl (V.S.-P.); 4 Institute of Computer Science, Faculty of Computer Science and Material Science, Silesian University ul. Bedzinska 39, 41-200 Sosnowiec, Poland; E-Mail: agnieszka.nowak@us.edu.pl

**Keywords:** Paralympic Games, paralympians, physical activity, sexual functioning

## Abstract

The purpose of this study was to determine sexual functioning of Polish athletes with disabilities (including paralympians). The study encompassed 218 people with physical disabilities, aged between 18 and 45 (149 men and 69 women). The entire research population was divided into three groups: Polish paralympians (n = 45), athletes with disabilities (n = 126) and non-athletes with disabilities (n = 47). The quality of sexual life of Polish paralympians was measured by using the Polish version of Female Sexual Function Index and International Index of Erectile Function. Clinically significant erectile dysfunctions were most often diagnosed in non-athletes (83.33%) with 50% result of severe erectile dysfunctions, followed by athletes and paralympians with comparable results of 56.98% and 54.17% respectively (*p* = 0.00388). Statistically significant clinical sexual dysfunctions concerned lubrication, orgasm as well as pain domains, and prevailed among female non-athletes (68.42%, 68.42% and 57.89%). Practising sports at the highest level has a favourable effect on the sexuality of men and women with physical disabilities. Men with physical disabilities manifest more sexual disorders than women, an aspect which should be considered by health-care professionals working with people with disabilities.

## 1. Introduction

The National Spinal Injuries Centre in Stoke Mandeville, UK, is considered the cradle of the Paralympic Games. In 1948 16 athletes with disabilities competed in the first National Stoke Mandeville Games for the Paralysed [1]. The first Summer Paralympic Games were held in Rome in 1960 under the name of the “First Games for the Disabled” and encompassed athletes with spinal cord injuries and other disabilities. Since 1960, every four years, the best athletes with disabilities have met at the largest sports competition for the disabled—the Paralympic Games [1,2].

Polish participants first competed at the 1972 Summer Paralympic Games held in Heidelberg, Germany. At present, the Polish paralympic team is composed of 94 competitors (60 men and 34 women). The participation in the Paralympic Games is a reward for years of immense effort and hard work. The competitors rely not only on their natural predispositions and sports talent, but they must also undergo special training, which is not substantially different from that of competitors without disabilities. This is a great challenge with significant health implications [3].

Human sexuality is a complex and multidimensional phenomenon. Many factors, including culture, social context, age, mental health, and interpersonal relations, may influence the sexual function of men and women. Physical disability may affect physical functioning, mood, the quality of life (QoL) and restrict sexual and non-sexual contacts. Yet, the data on the QoL and sexual functioning of men and women with physical disabilities is scarce, especially with regard to the correlations between physical activity and the abovementioned parameters [3,4,5,6]. 

Numerous studies have demonstrated that professional physical rehabilitation might significantly improve the QoL in men and women after spinal cord injury, which emphasizes the role of physical activity in the management of people with physical disabilities [4,5,6,7,8]. Knowing that the literature on disability (in particular spinal cord injury) repeatedly emphasizes the beneficial impact of regular physical activity on both physical and psychological health, and knowing that very few studies explored the beneficial effect of physical activity on sexual functions, sexual satisfaction and sexual well-being, exploring the relationship between various levels of physical activity, sexual function and satisfaction in individuals with disability might be a definitely innovative and interesting area, and is a unique contribution to the research [4,5,6,7,8].

The main purpose of this study was to determine the quality of sexual functioning of Polish athletes with disabilities (including paralympians). This is the first study showing the sexuality of Polish paralympians. The study offers important insights into the understanding of the association between physical disability and sexual functioning of Polish people with disabilities, and in particular of paralympians. The primary interest of the paper emphasizes more generally the importance of physical activity, both in terms of paralympians and athletes, and in some cases even in terms of physical rehabilitation.

## 2. Materials and Methods

### 2.1. Participants—Research Groups

The study encompassed 218 people with physical disabilities, aged between 18 and 45 (149 men and 69 women). The entire research population was divided into three groups: Polish paralympians (n = 45), athletes with disabilities (n = 126) and non-athletes with disabilities (n = 47).

The paralympians (Paralympians) were recruited from Polish sport clubs for individuals with disabilities. The inclusion criteria for the first group were: the membership of Polish sport clubs for people with disabilities, qualification for the Paralympic Games, consent to participation in the study, complete filling out of the questionnaire, general good health and age range of 18–45 years old. The original group counted 65 Polish athletes who qualified for the Paralympic Games. The final analysis encompassed 45 athletes—69.23% (28 men and 17 women) as the remaining 20 were excluded from the study since they did not meet all the inclusion criteria. The exclusion criteria for this and the other study groups were lack of consent to participate in the study (in this group n = 8; 40%), incomplete filling out of the questionnaire (n = 7; 35%) and an age under 18 or over 45 years old (n = 5; 25%). Sixteen individuals had a spinal cord injury (35.56%), the rest constituted athletes with different inherited or acquired disease (e.g., bone/muscle inherited diseases, phocomelia, states after surgical or accidental limb amputation) (Table 1).

The second group (Athletes) included 126 athletes with disabilities (95 men and 31 women) who were members of Polish sport clubs for people with disabilities but who did not qualify for the Paralympic Games. The inclusion criteria for the Athletes group were membership in Polish sport clubs for people with disabilities, regular physical activity, consent to participate in the study, complete filling out of the questionnaire, general good health and age range of 18–45 years old. Twenty nine athletes with disabilities had a spinal cord injury (23.02%), the rest were athletes with different diseases.

The third group (Non-athletes) consisted of 47 healthy non-athletes with physical disabilities (26 men and 21 women). The inclusion criteria for the Non-athletes group were sedentary life style, a minimum of one year time after regular active therapeutic rehabilitation, no actual regular physical activity, consent to participate in the study, complete filling out of the questionnaire, general good health and an age range of 18–45 years old. In this group 18 individuals had a spinal cord injury (38.30%), the rest were non-athletes with different diseases (Table 1). 

For the final analysis only healthy persons were qualified (for every study group); individuals with any comorbidities (e.g., diabetes mellitus, hypertension, any endocrinological diseases) were excluded at the beginning of the research. The research program was approved by the Bioethics Committee of the Medical University of Silesia in Katowice, Poland. Informed consents were obtained from all study participants.

### 2.2. Procedures

The research tool was a questionnaire voluntarily and anonymously completed by the respondents of the research groups. The questionnaire was composed of a general part concerning the socio-demographic conditions (age, marital status, education, occupational activity, physical activity), medical history, health problems, a part dedicated to physical disability (the reason of disability, diagnosis, form of locomotion) and a detailed part in the form of self-evaluation inventories: the Polish version of Female Sexual Function Index (FSFI) and International Index of Erectile Function (IIEF) evaluating female and male sexual functioning. 

### 2.3. Female Sexual Function Index (FSFI)

FSFI has been confirmed and clinically documented with regard to validity, sensitivity, reliability, internal consistency, stability and test-retest reliability in diagnosing disorders of sexual desire, arousal, orgasm as well as dyspareunia [9,10,11].

FSFI is composed of 19 items divided into six collective domains (subscales): I—sexual desire, II—sexual arousal, III—lubrication, IV—orgasm, V—sexual satisfaction and VI—dyspareunia. The final results are obtained separately for each of the subscales by summing up the elementary points encompassed within each of the 6 domains and a selected coefficient. The interpretation of partial results is a linear dependence: the higher the score, the better the sexual functioning within a given category [9,10,11]. The next stage is a global evaluation of the entire FSFI scale. Results below 65% of the maximum number of points scored in each of the domains (less than 3.9 points) were considered as sexual dysfunction in that domain. In a global FSFI assessment, clinically significant female sexual disorders (FSD) were diagnosed at values lower or equal to 26.55 points. Sexual disorders were diagnosed according to the American Psychiatric Association's Diagnostic and Statistical Manual of Mental Disorders Fourth Edition (DSM-IV) and the American Foundation for Urologic Disease (AFUD) criteria (scores of 26.55 or less on the FSFI and 3.9 or less in each of its domains, with the presence of sexual distress) [11].

### 2.4. International Index of Erectile Function (IIEF)

IIEF is a multidimensional, 5-grade instrument for self-evaluation of all male sexual functions within the previous 4 weeks [12,13,14,15]. It is characterised by high validity, reliability, sensitivity and test-retest reliability in the diagnosing of changes, confirmed by over 50 clinical trials [13,14]. The implementation of IIEF is a recommended standard in the diagnosis and evaluation of erectile dysfunctions and their intensification [13,15,16].

The IIEF questionnaire encompasses 15 items grouped in five collective domains (subscales) describing: I—erectile function, II—orgasm function, III—sexual desire, IV—intercourse satisfaction and V—overall satisfaction [12,13,15]. The total scores within all the domains (I–V) create a positive dependence with correct sexual functioning [12,15]. An additional analysis of the erectile subscale facilitates the isolation of four disorder intensification levels (Erectile Dysfunction—ED): erectile function (26–30 points), mild ED (17–25 points), moderate ED (11–16 points) and severe ED (6–10 points) [13]. Clinically significant erectile dysfunction is diagnosed at values equal to or less than 25 points (cut-off point) [13].

### 2.5. Statistical Analysis

STATISTICA 10.0 (StatSoft, Tulsa, OK, USA) for Windows was used in the statistical analysis. Differences among parameters were considered significant at the level of 0.05. The statistical analysis made use of: Shapiro-Wilk test, Mann-Whitney U-test, CHI^2^, Kruskal-Wallis covariance and *post-hoc* tests.

## 3. Results

### 3.1. Participants—Socio-Demographic Characteristics

All participants (100%) were Polish, White/Caucasian. In the first stage the statistical comparison of the three groups was performed (univariate analysis, Kruskal-Wallis/CHI^2^ test). Non-athletes with disabilities were significantly older than paralympians and athletes (mean age: 45.34 ± 15.93 *vs.* 34.10 ± 11.01 and 28.41 ± 10.37 years respectively) (*p* = 0.000001) (Table 1).

**Table 1:** Socio-demographic characteristics of the study population (mean ± SD**/**n (%)).

Variables		Paralympians	Athletes	Non-athletes	Kruskal-Wallis/CHI^2^ test
**Age (years)**		34.10 ± 11.01	28.41 ± 10.37	45.34 ± 15.93	*p* = 0.000001
**Residence**	Rural areas	9 (20.45%)	31 (24.80%)	15 (31.91%)	NS (*p* = 0.21221)
	Town <100,000	20 (45.45%)	38 (30.40%)	18 (38.30%)	
	Big city >100,000	15 (34.09%)	56 (44.80%)	14 (29.79%)	
**Marital status**	Single	28 (63.64%)	99 (80.49%)	34 (73.91%)	NS (*p* = 0.07902)
	Married	16 (36.36%)	24 (19.51%)	12 (26.09%)	
**Education**	Primary	1 (2.22%)	7 (5.56%)	3 (6.38%)	NS (*p* = 0.15863)
	Vocational	7 (15.56%)	23 (18.25%)	17 (36.17%)	
	Secondary	27 (60.00%)	69 (54.76%)	19 (40.43%)	
	Tertiary	10 (22.22%)	27 (21.43%)	8 (17.02%)	
**Occupational activity**	Unemployed	12 (27.27%)	27 (21.43%)	29 (61.70%)	*p* = 0.00002
	Employed	22 (50.00%)	59 (46.83%)	12 (25.53%)	
	Student	10 (22.73%)	40 (31.75%)	6 (12.77%)	
**The cause of physical disability**	Inherited	20 (44.44%)	55 (43.65)	6 (12.77%)	*p* = 0.00044
	Spinal cord injury	16 (35.56%)	29 (23.02%)	18 (38.30%)	NS (*p* = 0.37352)
	Disease	11 (24.44%)	16 (12.70%)	14 (29.79%)	*p* = 0.02447
**The form of locomotion**	Unaided	24 (23.76%)	65 (52.00%)	12 (26.09%)	*p* = 0.01300
	On crutches	9 (23.08%)	17 (13.60%)	13 (28.26%)	
	Wheelchair	10 (22.22%)	42 (33.60%)	18 (39.13%)	
	Others	2 (33.33%)	1 (0.80%)	3 (6.52%)	

SD—standard deviation; NS—not significant.

Generally, the research groups were statistically comparable with regard to the place of residence, marital status, education level, age at the first intercourse, frequency of sexual intercourse and number of sexual partners (Table 1 and Table 2). 

**Table 2:** Sexual behaviours in the study population (mean ± SD; %).

Variables		Paralympians	Athletes	Non-athletes	Kruskal-Wallis/CHI^2^ test
**Age at the first intercourse (years)**		19.51 ± 5.07	18.66 ± 3.75	18.38 ± 3.35	NS (*p* = 0.430479)
**Length of current relationship (years)**		8.45 ± 8.20	7.15 ± 7.89	16.57 ± 16.04	*p* = 0.002449
**The number of sexual partners**		8.75 ± 12.84	5.44 ± 6.93	4.27 ± 3.95	NS (*p* = 0.056261)
**Frequency of sexual intercourse (n, %)**	**once a day**	3 (8.11%)	5 (5.95%)	1 (3.85%)	NS (*p* = 0.53276)
	**several times/week**	14 (37.84%)	22 (26.19%)	4 (15.38%)	
	**several times/month**	11 (29.73%)	33 (39.29%)	12 (46.15%)	
	**1 or less/month**	9 (24.32%)	24 (28.57%)	9 (34.62%)	

SD—standard deviation; NS—not significant

Statistically significant differences concerned: occupational activity, physical disability and the form of locomotion as well as the length of current relationship (Table 1 and Table 2). The highest number of unemployed persons was found among non-athletes with disabilities (61.70%) (Table 1).

Among paralympians 16 individuals had a spinal cord injury (35.56%), the rest constituted athletes with different inherited or acquired disease (e.g., bone/muscle inherited diseases, phocomelia, states after surgical or accidental limb amputation). Twenty nine athletes with disabilities had a spinal cord injury (23.02%) and the rest different diseases. In the third group (non-athletes) 18 individuals had a spinal cord injury (38.30%), the rest were non-athletes with different diseases. The use of a wheelchair was the highest among non-athletes (39.13%), compared to athletes with disabilities and paralympians (33.60% *vs.* 22.22% respectively) (Table 1).

### 3.2. Sexual Functioning of Men and Women with Disabilities—IIEF and FSFI Scores

The holistic evaluation of the IIEF scale and its five collective domains showed statistically significant differences in the IIEF global score (0.000009), erectile function (0.000133), orgasm (0.000199), sexual desire (0.0000001), intercourse satisfaction (0.000012) and overall satisfaction (0.016920) between the groups. Male paralympians showed the best sexual functioning (Kruskal-Wallis covariance test) (Table 3). 

Additionally, applying the *post-hoc* analysis, statistically significant differences were observed mostly between non-athletes and athletes (*p* = 0.000046) as well as between non-athletes and paralympians (*p* = 0.000004) and included IIEF global score and all IIEF domains.

Clinically significant erectile dysfunctions were most often diagnosed in non-athletes (83.33%) with 50% result of severe erectile dysfunctions, followed by athletes and paralympians with comparable results of 56.98% and 54.17%, respectively (*p* = 0.00388) (Table 3).

**Table 3:** IIEF scores in studied men (mean ± SD; min–max; %).

IIEF domains				Paralympians	Athletes	Non-athletes		Kruskal-Wallis test	
**IIEF global score**			Mean ± SD	59.08 ± 11.14	51.05 ± 20.45	32.48 ± 21.80		0.000009	
			Min–Max	30–73	4–75	5–65			
**Erectile function**			Mean ± SD	23.75 ± 5.84	20.56 ± 9.39	12.82 ± 10.67		0.000133	
			Min–Max	6–30	0–30	1–29			
**Orgasm function**			Mean ± SD	8.29 ± 2.69	7.13 ± 3.25	4.54 ± 3.36		0.000199	
			Min–Max	2–10	1–10	1–10			
**Sexual desire**			Mean ± SD	8.58 ± 1.50	8.00 ± 1.95	5.40 ± 2.63		0.0000001	
			Min–Max	5–10	2–10	2–9			
**Intercourse satisfaction**			Mean ± SD	10.96 ± 2.39	8.70 ± 4.76	4.75 ± 4.67		0.000012	
			Min–Max	6–14	0–15	0–12			
**Overall satisfaction**			Mean ± SD	7.92 ± 1.69	7.51 ± 2.50	6.08 ± 2.62		0.016920	
			Min–Max	4–10	2–10	2–10			
**Erectile Dysfunction**		No ED n (%)		11 (45.83%)	37 (43.02%)	4 (16.67%)		*p* = 0.00388	
		Mild ED n (%)		11 (45.83%)	25 (29.07%)	7 (29.17%)			
		Moderate ED n (%)		1 (4.17%)	10 (11.63%)	1 (4.17%)			
		Severe ED n (%)		1 (4.17%)	14 (16.28%)	12 (50.00%)			

SD—standard deviation; NS—not significant; IIEF—International Index of Erectile Function; ED—Erectile Dysfunction.

Similar correlations were noticed in the evaluation of female sex life (FSFI test). The holistic evaluation of the FSFI scale and its six collective domains showed statistically significant differences in the FSFI global score (*p* = 0.008507), arousal (*p* = 0.047316), lubrication (*p* = 0.016709), orgasm (*p* = 0.004767), satisfaction (*p* = 0.025647) and pain (*p* = 0.007658). Female paralympians showed the best sexual functioning (Kruskal-Wallis covariance test) (Table 4). However, *post-hoc* analysis revealed that the differences concerned FSFI global score and its six domains were observed between the female paralympians and athletes (*p* = 0.010833) as well as between paralympians and non-athletes (*p* = 0.003391).

Implementing the cut-off points, statistically significant clinical sexual dysfunctions concerned lubrication, orgasm as well as pain domains, and prevailed among female non-athletes (68.42%, 68.42% and 57.89%). The global FSD were observed in athletes and non-athletes (65.38% and 63.16%); however, the values were not statistically significant (*p* = 0.13217) (Table 4). 

**Figure 1:**
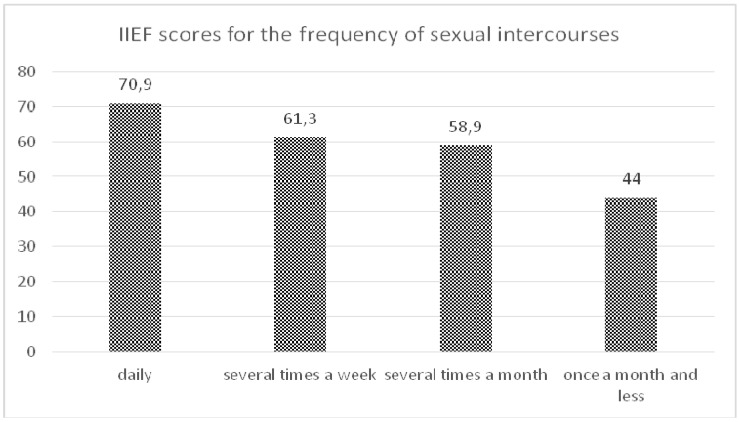
IIEF scores for the frequency of sexual intercourses in studied men.

The additional statistical analysis showed that the frequency of sexual intercourses statistically positively correlated only with IIEF global score (*p* = 0.0000001) (Figure 1). These differences with FSFI scores were not statistically significant. 

**Table 4:** FSFI scores and sexual dysfunctions in studied women (mean ± SD; min–max; %).

IIEF domains		Paralympians	Athletes	Non-athletes	Kruskal-Wallis test
FSFI global score	Mean ± SD	27.8 ± 7.22	18.42 ± 11.55	16.21 ± 12.48	*p* = 0.008507
	Min-Max	11.6–34.5	2–33.7	2.4–34.1	
Desire	Mean ± SD	3.96 ± 1.08	3.32 ± 1.32	3.19 ± 1.78	NS (*p* = 0.262703)
	Min-Max	1.8–6	0–5.4	0–6	
Arousal	Mean ± SD	4.34 ± 1.91	2.73 ± 2.38	2.53 ± 2.32	*p* = 0.047316
	Min-Max	0-6	0–6	0–5.4	
Lubrication	Mean ± SD	4.78 ± 2.01	3.01 ± 2.62	2.35 ± 2.45	*p* = 0.016709
	Min-Max	0–6	0–6	0–6	
Orgasm	Mean ± SD	4.88 ± 1.44	2.75 ± 2.42	2.46 ± 2.42	*p* = 0.004767
	Min-Max	0–6	0–6	0–6	
Satisfaction	Mean ± SD	4.8 ± 0.93	3.84 ± 2.01	2.97 ± 2.27	*p* = 0.025647
	Min-Max	3.2–6	0–6	0–6	
Pain	Mean ± SD	5.04 ± 1.61	3.06 ± 2.78	2.42 ± 2.36	*p* = 0.007658
	Min-Max	0–6	0–6	0–6	
Female sexual dysfunctions	Desire disorders n (%)	7 (46.67%)	19 (73.08%)	13 (68.42%)	NS (*p* = 0.21660)
	Arousal disorders n (%)	4 (26.67%)	15 (57.69%)	11 (57.89%)	NS (*p* = 0.11328)
	Lubrication disorders n (%)	2 (13.33%)	14 (53.85%)	13 (68.42%)	*p* = 0.00464
	Orgasmic disorders n (%)	1 (6.67%)	16 (61.54%)	13 (68.42%)	*p* = 0.00049
	Satisfaction disorders n (%)	3 (20.00%)	8 (30.77%)	10 (52.63%)	NS (*p* = 0.14183)
	Pain disorders n (%)	1 (6.67%)	12 (46.15%)	11 (57.89%)	*p* = 0.00712
	Global FSD n (%)	5 (33.33%)	17 (65.38%)	12 (63.16%)	NS (*p* = 0.13217)

SD—standard deviation; NS—not significant; FSFI—Female Sexual Functioning Index.

The form of locomotion also positively correlated with IIEF. Males on wheelchair revealed the worst sexual functioning (*p* = 0.048173). Both employed men and women showed the best sexual functioning evaluated by the IIEF and FSFI scales (*p* = 0.04512 and *p* = 0.008992 respectively). 

Marital status also had a significant effect on sexuality among research population. Both married men and women obtained statistically higher mean general scores in IIEF and FSFI scales (IIEF: 56.41 ± 17.62 and FSFI: 26.24 ± 7.63; *p* = 0.025046 and *p* = 0.023436 respectively). 

## 4. Discussion

There are numerous studies on the QoL, psychological aspects and sexuality of people with disabilities after spinal cord injury. Some studies focus on the effect of physical activity in the form of rehabilitation on general well-being [4,5,6,7,8,17,18,19,20,21,22,23,24,25,26]. However, there is a paucity of data regarding a specific group of athletes with disabilities who practice sports at the highest level—the paralympians. Because of the different study groups and the considerably different levels of physical activity in our research groups, a comparison of our results with those of other studies could prove ambiguous. 

Our study is one of the first on the subject, and offers unique findings on this important issue. We showed the comparison of individuals with disabilities depending on the level of physical activity. We found that the higher level of physical activity the better sexual functions in people with disabilities. However, individuals who practice sports at the highest level—the paralympians/athletes with disabilities (in our study) generally do not differ. It might be hypothesized that there is a threshold above which physical activity no longer improve sexual functioning.

However, human sexuality is a complex and multidimensional phenomenon and many additional factors, including culture, social context, age, mental health, and interpersonal relations, may influence the sexual function of men and women. Therefore, the interpretation of our results should be careful. It was difficult to isolate additional factors affecting personal sexuality among study participants.

Medical publications report that persons with spinal cord injury demonstrate a series of disabilities and limitations (including: general physical health, QoL, psychological functioning, social and personal relations as well as sex life) [4,17,18]. These results are consonant only with our third group—non-athletes with disabilities. Therefore, it might be suggested that regular physical activity can improve QoL.

Numerous studies demonstrate that professional medical help, especially physical rehabilitation, might significantly improve physical and mental well-being as well as sexual functions in men and women after spinal cord injury, which emphasizes the special role of physical activity in the management of people with physical disabilities [4,5,6,7,8].

Spinal cord injuries can have a significant impact on sexual functioning. The majority of clinical papers indicate that spinal cord injury affects particularly men’s sexual behaviour in terms of sexual performance and body sensitivity [6,7,19,20,21,22]. The studies also reveal that physical disability can impair the psychological and physical aspects of female sexual arousal. However, most results demonstrate that the sex life of women with spinal cord injury remains less affected than among men [5,8,23,24,25,26,27,28]. 

Our findings are comparable. The female clinical sexual dysfunctions concerned only lubrication, orgasm and pain, and prevailed in female non-athletes. The percentage of FSD did not differ significantly between our study groups. By contrast, clinically significant ED were diagnosed quite frequently (non-athletes—83.33%, athletes—56.98% and paralympians—54.17%). It is well-known that female sexuality is mostly depended on mental well-being and interpersonal relations.

According to international societies of sexual medicine, the direct and indirect effects of chronic diseases (including physical disability) on sexual health are frequent and complex. Nevertheless, there are no specific guidelines for their optimal management. Therefore, further research and scientific reporting on the prevalence, pathophysiology and optimal treatment of sexual dysfunction associated with chronic illness is needed [29].

In 2010, the Consortium for Spinal Cord Medicine published “*Sexuality and Reproductive Health in Adults with Spinal Cord Injury: A Clinical Practice Guideline for Health-Care Professionals*” with a view to encourage individuals to take an active role in obtaining information related to sexual issues as well as encourage people with spinal cord injury to explore the role of sexuality in their lives. The professionals suggest developing a sexual education and treatment plan with the individual consistent with the results of the sexual history, interview, relationship status and physical exam findings. Sexual information and counselling should be available both during initial rehabilitation and later as a follow-up when the persons with disabilities have returned to their homes [30].

Currently, there are only two articles in the PubMed database evaluating sportsmen with disabilities, and they do not make any reference to paralympians. Tasiemski *et al.*, in their study, examined the interrelationships among athletic identity, sport participation and psychological adjustment in a sample of people with spinal cord injury. The authors concluded that being able to practice one’s favourite sport after injury was associated with higher levels of athletic identity and better psychological adjustment. Team sport participants reported experiencing better psychological adjustment than individual sport participants [31].

Dinomais *et al.* investigated social functioning, quality of life and self-esteem in 496 young athletes with disabilities taking part in adapted competitive sports. The researchers noticed significantly higher social functioning scores in this population, which confirms the positive effect of sport on the general well-being of physically people with disabilities [32].

The design of our study offers important insights into the understanding of the association between physical disability and sexual functioning of Polish athletes with disabilities, and in particular of paralympians. It presents potentially valuable implications for health care professionals working with people with physical disabilities. Firstly, by including a numerous group of Polish paralympians (66.15%), we endeavoured to ensure reliable results. Secondly, the use of self-reporting in evaluating sexual functioning encouraged the participants to express openly the majority of their problems. Thirdly, this is one of the first clinical research evaluating sex life in such a specific group of sportsmen with disabilities as the paralympians.

Despite all these advantages, the limitations of the study must also be recognized. Firstly, the study sample may be too small to generalize the obtained results to the entire population of Polish people with physical disabilities. Secondly, individuals who were particularly uncomfortable talking about their sex/intimate life may have been less likely to respond truthfully. Thirdly, non-athletes more often used wheelchairs as compared with athletes and paralympians; moreover, the largest percentage of persons with spinal cord injury was found among these groups which might negatively affect their sexuality. Fourthly, we did not access the detailed information about spinal cord injury levels and severity (e.g., ASIA scores, medical history). The data only based on questionnaires responses. Finally, the authors did not concentrate on non-intercourse sexual activity (e.g., fellatio, cunnilingus), which is described as a major component of routine sexual activity among individuals with disabilities. This fact might affect the results of the present study. Moreover, the authors did not isolate additional factors effecting personal sexuality among study participants. Therefore, the interpretation of results concerning sexuality should be careful. 

## 5. Conclusions

The analysis of our research material shows that practising sports at the highest level has a favourable effect on the sexuality of men and women with physical disabilities. Men with physical disabilities manifest more sexual disorders than women, an aspect which should be considered by health-care professionals working with people with disabilities. Future research in which these associations are examined longitudinally is clearly warranted, especially with a view to indicate groups at risk of developing sexual disturbances. Accurate screening for sexual and psychological problems in every person with a physical disability is strongly recommended. 

## References

[1] Silver J.R. (2004). The role of sport in the rehabilitation of patients with spinal injuries. J. R. Coll. Phys. Edinb..

[2] Gold J.R., Gold M.M. (2007). Access for all: The rise of the Paralympic Games. J. R. Soc. Promot. Health.

[3] Sobiecka J., Plinta R., Drobniewicz K., Kłodecka-Różalska K., Cichoń K. (2012). Conditions for preparations for the 2008 Beijing Paralympic Games in the opinion of the Polish national team. Biomed. Hum. Kinet..

[4] Alexander M.S., Brackett N.L., Bodner D., Elliott S., Jackson A., Sonksen J., National Institute on Disability and Rehabilitation Research (2009). Measurement of sexual functioning after spinal cord injury: Preferred instruments. J. Spin. Cord Med..

[5] Lombardi G., Del Popolo G., Macchiarella A., Mencarini M., Celso M. (2010). Sexual rehabilitation in women with spinal cord injury: A critical review of the literature. Spin. Cord..

[6] Fisher T.L., Laud P.W., Byfield M.G., Brown T.T., Hayat M.J., Fiedler I.G. (2002). Sexual health after spinal cord injury: A longitudinal study. Arch. Phys. Med. Rehabil..

[7] Dahlberg A., Alaranta H.T., Kautiainen H., Kotila M. (2007). Sexual activity and satisfaction in men with traumatic spinal cord lesion. J. Rehabil. Med..

[8] Kreuter M., Siösteen A., Biering-Sørensen F. (2008). Sexuality and sexual life in women with spinal cord injury: A controlled study. J. Rehabil. Med..

[9] Rosen R., Brown C., Heiman J., Leiblum S., Meston C., Shabsigh R., Ferguson D., D’Agostino R. (2000). The Female Sexual Function Index (FSFI): A multidimensional self-report instrument for the assessment of female sexual function. J. Sex Marit. Ther..

[10] Meston C.M. (2003). Validation of the Female Sexual Function Index (FSFI) in women with female orgasmic disorder and in women with hypoactive sexual desire disorder. J. Sex Marit. Ther..

[11] Ferenidou F., Kapoteli V., Moisidis K., Koutsogiannis I., Giakoumelos A., Hatzichristou D. (2008). Presence of a sexual problem may not affect women’s satisfaction from their sexual function. J. Sex Med..

[12] Rosen R.C., Riley A., Wagner G., Osterloh I.H., Kirkpatrick J., Mishra A. (1997). The International Index of Erectile Function (IIEF): A multidimensional scale for assessment of erectile dysfunction. Urology.

[13] Cappelleri J.C., Rosen R.C., Smith M.D., Mishra A., Osterloh I.H. (1999). Diagnostic evaluation of the erectile function domain of the International Index of Erectile Function. Urology.

[14] Rosen R.C., Cappelleri J.C., Smith M.D., Lipsky J., Peña B.M. (1999). Development and evaluation of an abridged, 5-item version of the International Index of Erectile Function (IIEF-5) as a diagnostic tool for erectile dysfunction. Int. J. Impot. Res..

[15] Rosen R.C., Cappelleri J.C., Gendrano N. (2002). The International Index of Erectile Function (IIEF): A state-of-the-science review. Int. J. Impot. Res..

[16] Lue T.F., Giuliano F., Montorsi F., Rosen R.C., Andersson K.E., Althof S., Christ G., Hatzichristou D., Hirsch M., Kimoto Y., Lewis R., McKenna K., MacMahon C., Morales A., Mulcahy J., Padma-Nathan H., Pryor J., de Tejada I.S., Shabsigh R., Wagner G. (2004). Summary of the recommendations on sexual dysfunctions in men. J. Sex Med..

[17] Vall J., Costa C.M., Pereira L.F., Friesen T.T. (2011). Application of International Classification of Functioning, Disability and Health (ICF) in individuals with spinal cord injury. Ar. Neuropsiquiatr..

[18] Reitz A., Tobe V., Knapp P.A., Schurch B. (2004). Impact of spinal cord injury on sexual health and quality of life. Int. J. Impot. Res..

[19] Abramson C.E., McBride K.E., Konnyu K.J., Elliott S.L., SCIRE Research Team (2008). Sexual health outcome measures for individuals with a spinal cord injury: A systematic review. Spin. Cord.

[20] Anderson K.D., Borisoff J.F., Johnson R.D., Stiens S.A., Elliott S.L. (2007). The impact of spinal cord injury on sexual function: Concerns of the general population. Spin. Cord.

[21] Deforge D., Blackmer J., Garritty C., Yazdi F., Cronin V., Barrowman N., Fang M., Mamaladze V., Zhang L., Sampson M., Moher D. (2006). Male erectile dysfunction following spinal cord injury: A systematic review. Spin. Cord.

[22] Cardoso F.L., Savall A.C., Mendes A.K. (2009). Self-awareness of the male sexual response after spinal cord injury. Int. J. Rehabil. Res..

[23] Ferreiro-Velasco M.E., Barca-Buyo A., de la Barrera S.S., Montoto-Marqués A., Vázquez X.M., Rodríguez-Sotillo A. (2005). Sexual issues in a sample of women with spinal cord injury. Spin. Cord.

[24] Anderson K.D., Borisoff J.F., Johnson R.D., Stiens S.A., Elliott S.L. (2007). Spinal cord injury influences psychogenic as well as physical components of female sexual ability. Spin. Cord.

[25] Kreuter M., Taft C., Siösteen A., Biering-Sørensen F. (2011). Women’s sexual functioning and sex life after spinal cord injury. Spin. Cord.

[26] Matzaroglou C., Assimakopoulos K., Panagiotopoulos E., Kasimatis G., Dimakopoulos P., Lambiris E. (2005). Sexual function in females with severe cervical spinal cord injuries: A controlled study with the Female Sexual Function Index. Int. J. Rehabil. Res..

[27] Komisaruk B.R., Gerdes C.A., Whipple B. (1997). ‘Complete’ spinal cord injury does not block perceptual responses to genital self-stimulation in women. Arch. Neurol..

[28] Sipski M.L., Alexander C.J., Rosen R.C. (1997). Physiologic parameters associated with sexual arousal in women with incomplete spinal cord injuries. Arch. Phys. Med. Rehabil..

[29] Basson R., Rees P., Wang R., Montejo A.L., Incrocci L. (2010). Sexual function in chronic illness. J. Sex Med..

[30] Consortium for Spinal Cord Medicine (2010). Sexuality and reproductive health in adults with spinal cord injury: A clinical practice guideline for health-care providers. J. Spin. Cord Med..

[31] Tasiemski T., Brewer B.W. (2011). Athletic identity, sport participation, and psychological adjustment in people with spinal cord injury. Adapt. Phys. Activ. Q..

[32] Dinomais M., Gambart G., Bruneau A., Bontoux L., Deries X., Tessiot C., Richard I. (2010). Social functioning and self-esteem in young people with disabilities participating in adapted competitive sport. Neuropediatrics.

